# Aggressive Resection for a Primary Leiomyosarcoma of the Vena Cava Masquerading as a Pancreatic Head Tumor

**DOI:** 10.7759/cureus.46634

**Published:** 2023-10-07

**Authors:** Shamir O Cawich, Dave Harnanan, Adrian Coye, Lisa J Johnson, Hugh E Sanchez, Tracey Nicholas

**Affiliations:** 1 Surgery, University of the West Indies, St. Augustine, TTO; 2 Clinical Surgical Sciences, The University of the West Indies, St. Augustine, TTO; 3 Cardiothoracic Surgery, Karl Heusner Memorial Hospital, Belize City, BLZ; 4 Surgery, University of Belize, Belize City, BLZ; 5 Pathology, Karl Heusner Memorial Hospital, Belize City, BLZ; 6 Surgery, Karl Heusner Memorial Hospital, Belize City, BLZ

**Keywords:** vascular, primary, leiomyosarcoma, vena cava, resource-poor

## Abstract

Smooth muscle is a normal component of the inferior vena cava (IVC) wall. Although uncommon, the smooth muscle component may undergo neoplastic change. Benign neoplasms are termed leiomyomas, and when there is a malignant change, the nomenclature is changed to an IVC leiomyosarcoma.

Leiomyosarcomas of the IVC are rare, with less than 150 cases reported in medical literature. Unfortunately, the majority of IVC leiomyosarcomas are diagnosed at advanced disease stages. Surgical resection of locally advanced lesions is technically challenging, but complete resection is the mainstay of treatment as leiomyosarcomas respond poorly to chemo-radiotherapy.

Due to the advanced disease stage at diagnosis and the technical complexity of IVC resection and reconstruction, most patients are transferred to high-volume centers in developed nations. We report a case of a patient with a locally advanced leiomyosarcoma masquerading as a pancreatic head tumor. This patient could not access care in a high-volume center and required aggressive maneuvers to resect the IVC leiomyosarcoma in a resource-poor, low-volume center.

We present this case to highlight the steps in operative management and also to show that these procedures can be carried out in resource-poor environments once there is meticulous planning, appropriate equipment, and multidisciplinary care.

## Introduction

Primary sarcomas of the inferior vena cava (IVC) are rare [1-3]. Complete resections are the best therapeutic option since they respond poorly to other forms of therapy such as chemotherapy or radiotherapy [1-3]. Resection, however, is often difficult as they present late when the disease is locally advanced.

Due to the complexity of these operative procedures, these operations are often performed in high-volume centers. We report a case, where a patient underwent resection of a locally advanced primary leiomyosarcoma of the IVC in a low-resource setting.

## Case presentation

**Figure 1:**
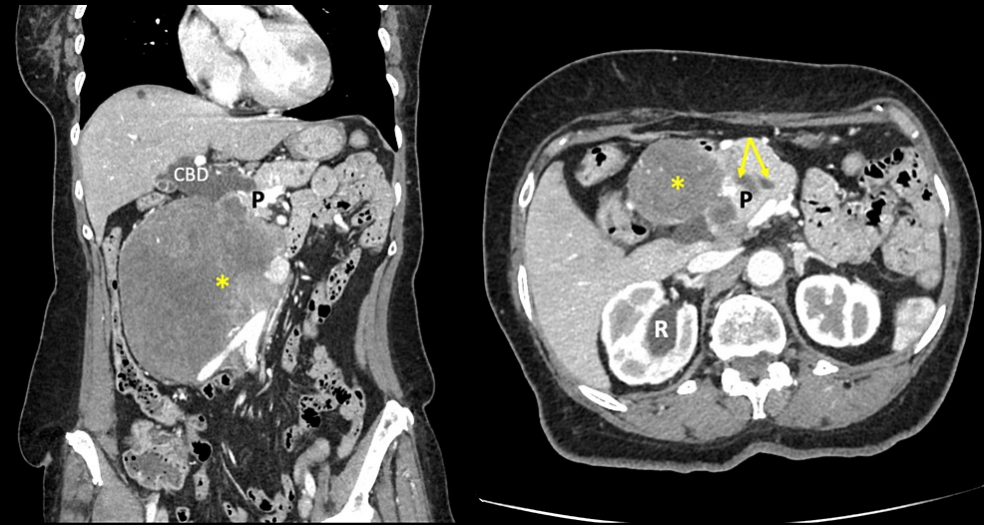
A contrast-enhanced CT scan of the abdomen in coronal (left) and corresponding axial (right) views. The large tumor (asterix) appears intimately related to the pancreatic head and uncinate process (P). In the axial views, a double duct sign (arrows) can be seen. The common duct is markedly dilated (CBD). There is also right hydronephrosis (R) on the axial images due to obstruction by the mass.

A 78-year-old woman with diabetes and hypertension presented to the hospital with a complaint of right flank pain. Upon examination, the abdomen was asymmetric due to the presence of a large, fixed mass in the right flank. A contrast-enhanced computerized tomography scan confirmed the presence of a 12x16cm lobulated mass in the retro-peritoneum. The mass was intimately related to the uncinate process of the pancreas but did not involve the superior mesenteric artery or vein (Figure 1). A double duct sign was present, suggestive of a peri-ampullary malignancy (Figure 1). There was involvement of the right ureter, causing right hydroureter and hydronephrosis (Figure 1).

**Figure 2:**
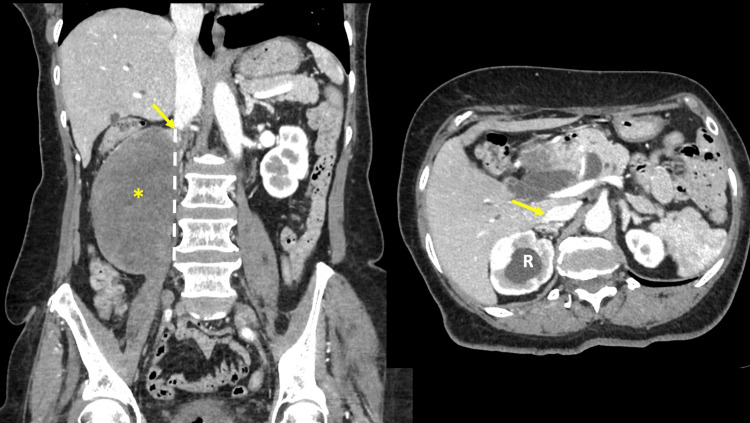
A contrast-enhanced CT scan of the abdomen in coronal (left) and corresponding axial (right) views. There is complete obliteration of the IVC at the level of the mass (broken line). The IVC reconstitutes at the retro-hepatic IVC (arrows). Hydronephrosis is again visible at the right kidney (R).

**Figure 3:**
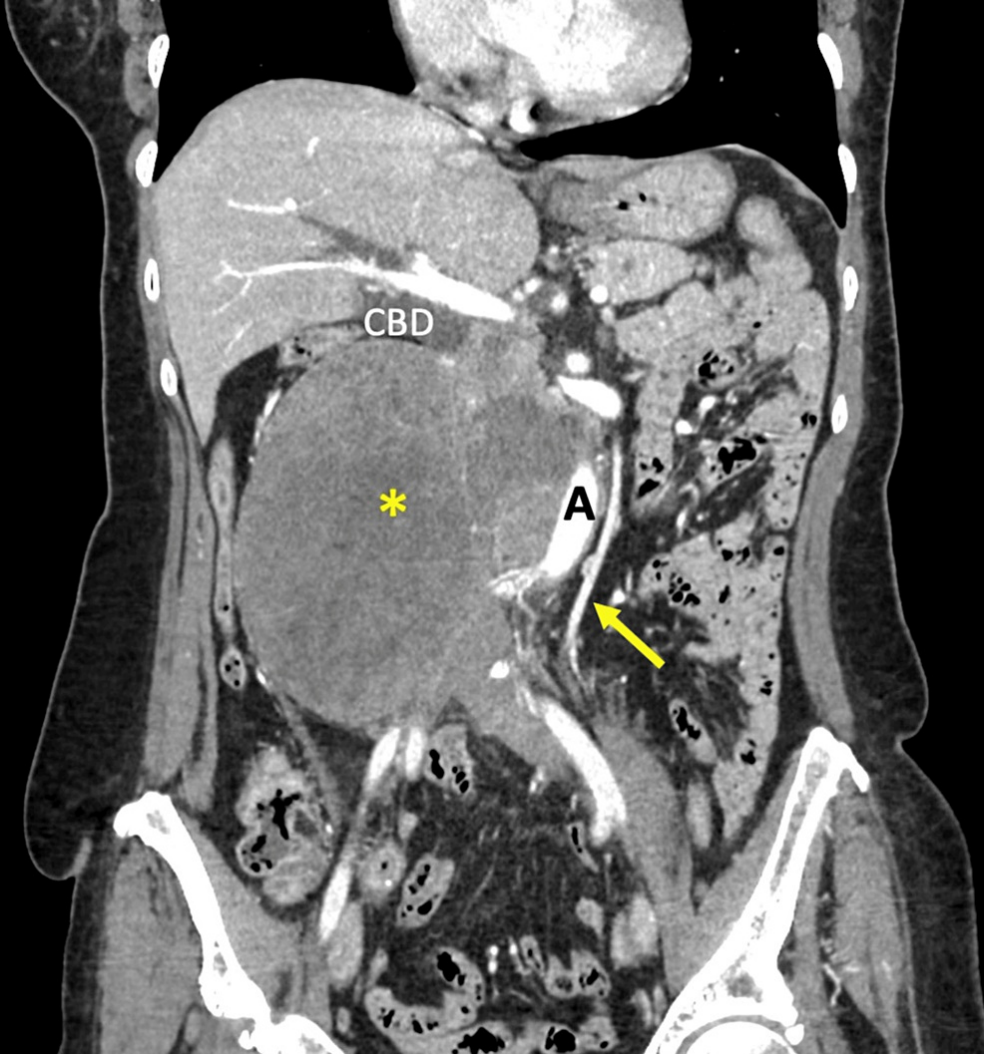
Coronal CT images The large mass (asterix) displaces the abdominal aorta (A) and superior mesenteric vessels (arrow) to the left. Dilatation of the common bile duct (CBD) is visible.

The mass appeared to compress the IVC, with downstream dilatation of the IVC and reconstitution at the retro-hepatic IVC (Figure 2). The abdominal aorta and mesenteric vessels were displaced to the left by the large mass (Figure 3). The stomach and duodenum were uninvolved in the endoscopic evaluation.

**Figure 4:**
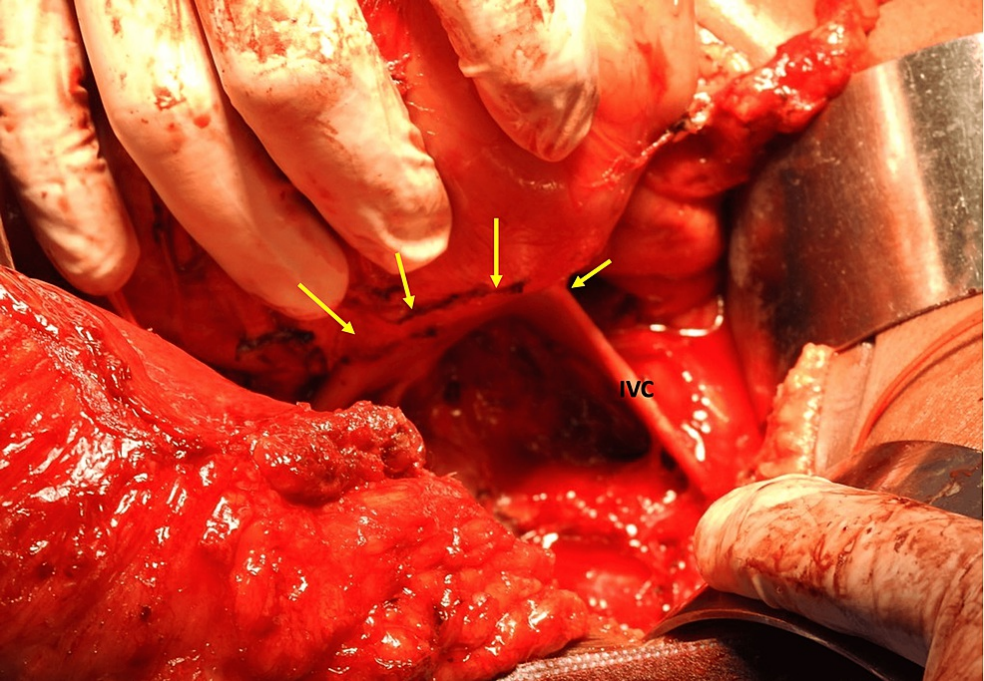
Intra-operative photograph of the retroperitoneum The mobilized mass is being lifted by the surgeon’s hand. The point of IVC invasion can be seen anteriorly (arrows)

**Figure 5:**
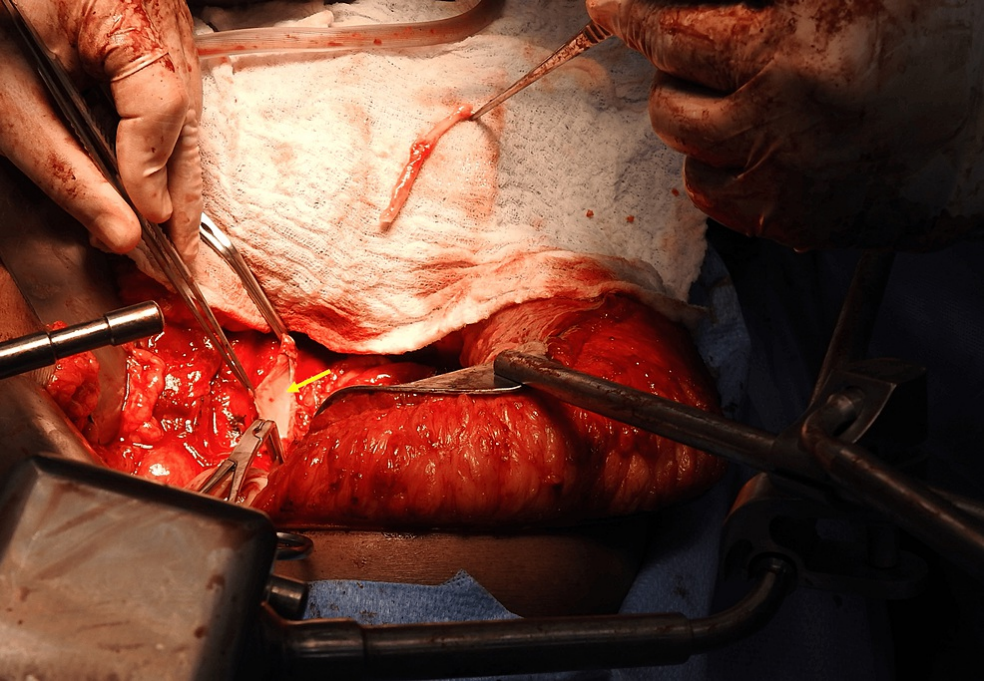
Operative photo with occlusive vascular clamps applied to the inferior vena cava The mass has been removed en bloc with a section of the caval wall. The arrows point to the IVC intima that is now visible through the defect in the caval wall.

**Figure 6:**
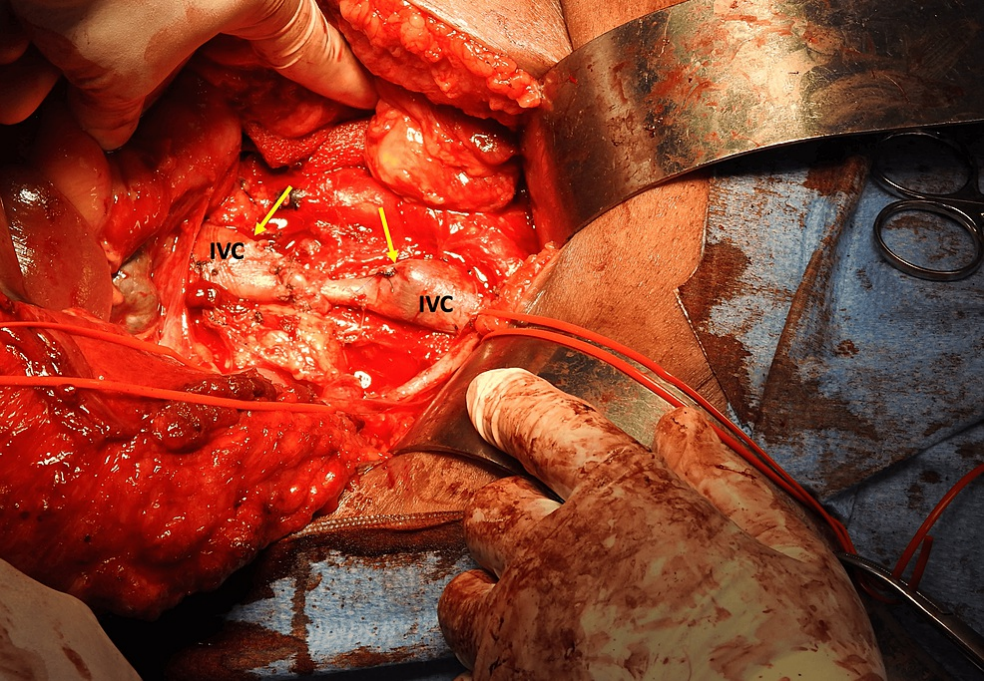
Operative photo after an attempt at primary repair of the inferior vena cava (IVC) Primary repair resulted in un-acceptable narrowing of the caval diameter to less than 50% of its normal diameter (arrows)

This patient was taken to the operating room for exploration and the surgical team was prepared to perform an extended Whipple’s operation with possible vascular resection and reconstruction, right nephrectomy and/or reconstruction of the IVC and right iliac vessels. The abdomen was opened using a modified Maccuchi incision. The large mass in the retroperitoneum was noted and there were no peritoneal metastases present. The right colon was mobilized medially to expose the duodenum and pancreatic head. A Kocher’s maneuver was performed to lift the pancreatic head and duodenum from the mass (Figure 4). The small bowel mesentery and the right ureter were mobilized from the retroperitoneal mass. The mass was mobilized infero-laterally and was noted to be separate from the right kidney. After complete mobilization, the mass appeared to be intimately related to the infra-renal IVC (Figure 5). A decision was taken to cross-clamp the IVC and resect the mass en-bloc with a cuff of IVC (Figure 6).

**Figure 7:**
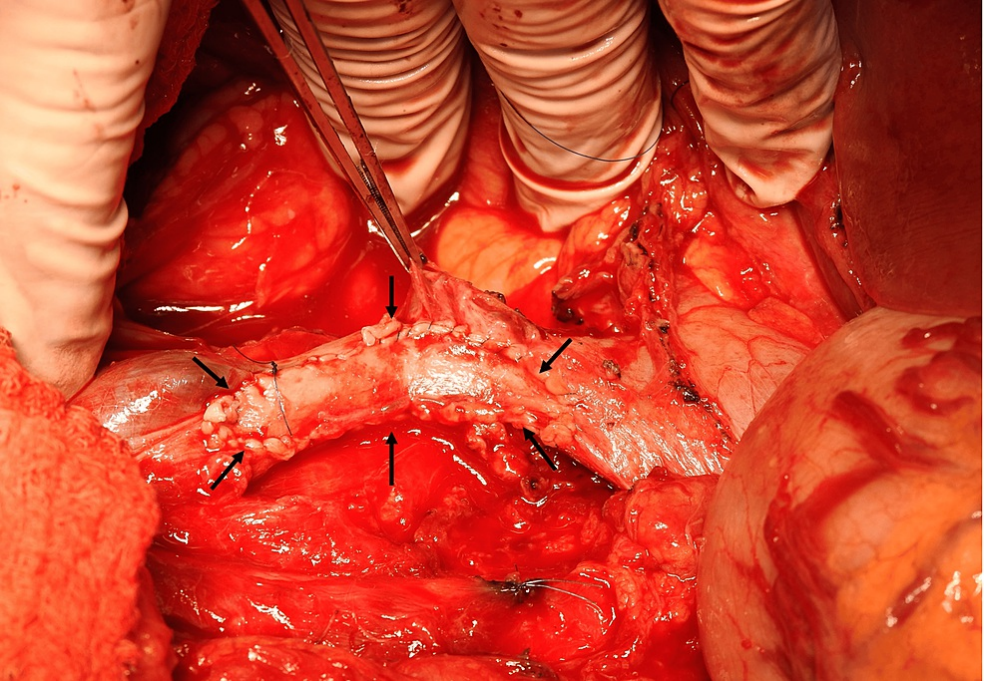
Reconstruction of the IVC The ipsilateral saphenous vein has been harvested and continuous sutures were used to secure it as a patch onto the IVC defect to reconstruct the IVC (arrows). This resulted in acceptable vein diameter post-reconstruction.

**Figure 8:**
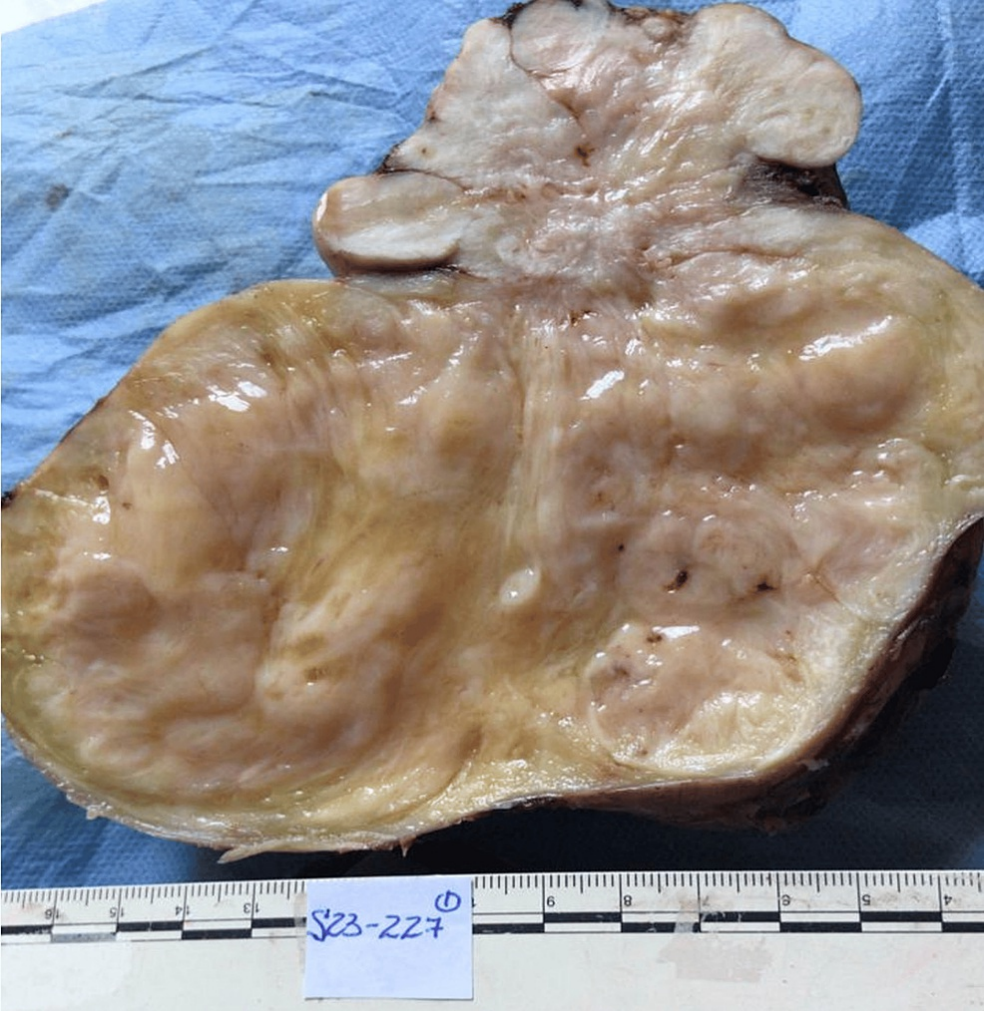
Gross pathologic specimen Large bosselated mass with tissues arranged in whorls and with interlacing fascicles, reminiscent of a leiomyoma

An attempt at primary IVC repair resulted in a narrowing of the caval diameter by more than 50% (Figure 7). The surgical team were not willing to accept this. Therefore, the ipsilateral great saphenous vein was harvested and prepared. Subsequently, the IVC was cross-clamped a second time and a saphenous vein patch was sutured onto the defect to reconstruct the IVC and restore its diameter (Figure 8). A passive blake drain was left at the operative bed. This patient recovered well. She spent 24 hours in the ICU and a further four days on the surgical wards. She was discharged from the hospital without event.

Pathologic examination revealed an 8x12x14cm firm, bosselated mass weighing 800 grams, with a portion of IVC wall attached. On histologic assessment, the mass was composed of spindle cells arranged in whorls and with interlacing fascicles, reminiscent of a leiomyoma. The mass was further distinguished by bizarre, atypical cells with enlarged hyperchromatic nuclei, tumor giant cells and low mitotic activity. The overall histological picture was that of a well-differentiated primary IVC leiomyosarcoma.

## Discussion

Primary IVC sarcomas are rare lesions. To the best of our knowledge only small case series [1-8] have been reported, with the largest published series reporting on only 22 cases [2]. We have encountered no reports from Belize, where this case originated, or the wider English-speaking Caribbean. Interestingly, the literature suggests that IVC leiomyosarcomas has a propensity of occur in middle-aged women with a 3:1 ratio [2], as in our index case.

In our patient, the diagnosis was made after she complained of ipsilateral flank pain. Interestingly, this was the most common symptom reported by patient in the series from Kieffer et al. [2,8]. The other findings of hydronephrosis and complete occlusion of the IVC were commonly reported in the literature [2,3,8]. Initially our pre-operative working diagnosis was a peri-ampullary malignancy, due to the presence of the double-duct sign. These findings are highly suggestive of peri-ampullary malignancies [9]. The diagnosis in our case was only made intra-operatively.

Although operative resection is the mainstay of treatment, these lesions usually require extensive resections as they are often diagnosed late when the lesion is locally advanced [1-8]. These aggressive resections carry a high operative risk, and in excess of 20% operative mortality when patients underwent aggressive resections [1-3,6,7].

In our case, in order to achieve wide resection margins, the IVC was crossed clamped at the infra-renal level (IVC segment I). This is standard procedure in these resections [1,5]. In our case, the IVC was clamped and occluded for approximately 10 minutes, but many authorities report that the IVC can be safely occluded for a period of up to 28 [2,10] to 40 minutes [11]. Some authors recommend simultaneously cross-clamping the infra-renal aorta when there is problematic arterial hypotension or proximal venous hypertension [2].

Prosthetic polytetraflouroethylene grafts may be used when required [2,12-14], and is accompanied by 10% incidence of graft thrombosis at a mean of 36 months follow up. In our case, we resected 2cm macroscopically normal caval margins in keeping with oncologic principles, and we only recognized that there would be unacceptable narrowing post-resection. We chose saphenous vein for patch angioplasty because prosthetic grafts were not universally available in our resource poor center, and we anticipated potential enzymatic leak as the pancreatic head and duodenum were extensively handled and mobilized.

In cases where IVC reconstruction is not possible, some authors recommend ligation of the IVC since there is still sufficient venous return through collaterals in the genital, azygos and lumbar veins [2]. Lower limb oedema is reported to occur only when proximal caval pressures exceed 30mmHg [2].

In cases where the sarcoma involves the retro-hepatic IVC (segment II), vascular exclusion of the liver is necessary, and can be achieved by using a Pringle manuever and clamping the IVC above and below the liver [2,15]. In cases where there is intra-cardiac extension of the sarcoma (segment III), cardiopulmonary bypass with circulatory arrest may be indicated [16,17].

Historically, primary IVC leiomyosarcomas were believed to be insensitive to chemotherapy and radiotherapy [1,3,6,18], but adjuvant chemotherapy is now considered appropriate after complete resection in modern oncology circles [1,2,19]. Hines et al. [4] also suggested that there is an emerging role for post-operative irradiation to infra-renal leiomyosarcomas of the IVCs. Due to the rarity of this diagnosis, there is little literature on long-term survival outcomes after complete resection. The literature reports that patients who undergo radical surgery with curative intent have overall survival ranging from 33% [4] to 53% [2,4] at five years. It is noteworthy that the overall survival is still better than patients who do not have complete resection, with median survival measured in months [2]. Therefore, there is consensus in the literature that aggressive resections are the best form of treatment for these lesions.

## Conclusions

Malignant neoplasms of the smooth muscle in the wall of the IVC are known as primary leiomyosarcomas. These are rare malignancies, with less than 150 reports of these lesions in the medical literature. They tend to produce vague symptoms and, therefore, are usually locally advanced at diagnosis.

Primary resection is the best therapeutic option for IVC leiomyosarcomas because the response to chemo-radiotherapy is poor. Resection and subsequent reconstruction of the IVC is technically challenging. Therefore, most authorities recommend that these operations should be done in high-volume centers.

We have reported a case of an IVC leiomyosarcoma that will add to the existing knowledge of this pathology. We also show that these major operations can be performed safely in expert hands at low-volume centers once there is meticulous planning, appropriate equipment, multidisciplinary cooperation, and expertise in vascular anastomoses.
